# Transcriptional Output Transiently Spikes Upon Mitotic Exit

**DOI:** 10.1038/s41598-017-12723-7

**Published:** 2017-10-03

**Authors:** Viola Vaňková Hausnerová, Christian Lanctôt

**Affiliations:** 1 BIOCEV and Department of Cell Biology, Faculty of Science, Charles University, Vestec, 252 50 Czech Republic; 2 First Faculty of Medicine, Charles University, Prague, 121 08 Czech Republic

## Abstract

The pulsatile nature of gene activity has recently emerged as a general property of the transcriptional process. It has been shown that the frequency and amplitude of transcriptional bursts can be subjected to extrinsic regulation. Here we have investigated if these parameters were constant throughout the cell cycle using the single molecule RNA FISH technique. We found evidence of transcriptional spikes upon mitotic exit in three different human cell lines. Recording of cell growth prior to hybridization and immuno-RNA FISH analysis revealed that these spikes were short-lived and subsided before completion of cytokinesis. The transient post-mitotic increase in transcriptional output was found to be the result of cells displaying a higher number of active alleles and/or an increased number of nascent transcripts per active allele, indicating that both the burst fraction and the amplitude of individual bursts can be increased upon mitotic exit. Our results further suggest that distinct regulatory mechanisms are at work shortly after mitotic exit and during the rest of interphase. We speculate that transcriptional spikes are associated with chromatin decondensation, a hallmark of post-mitotic cells that might alter the dynamics of transcriptional regulators and effectors.

## Introduction

Single cell studies revealed that transcription of most genes is a discontinuous process, with periods of activity interspersed with periods of inactivity^1^. This property, referred to as transcriptional bursting (or pulsing), helps to explain the cell-to-cell variability in the distribution of mRNA counts that is often observed in isogenic cell populations^2^. The pulsatile nature of transcription has been observed in a broad range of organisms, from *E. coli* to mammalian cells, albeit to a varying extent^3–5^. Elegant studies using GFP-based reporters succeeded in imaging transcriptional pulsing in living prokaryotic and eukaryotic cells^3,6,7^.

The cause of transcriptional pulsing remains unclear. Stochastic binding of transcription factors, supercoiling levels and chromatin structure have all been suggested to play determining roles^8–10^. Transcriptional kinetics and expression noise have also been correlated with promoter architecture. For instance, engineering changes in the binding affinity of *cis*-acting sequences for their cognate factors or modulating the concentration of these factors can lead to changes in the rate of promoter activation (burst fraction) and in the number of transcripts produced during ‘on’ periods (burst duration)^11–13^. The biological significance of the discontinuous nature of transcription is unclear. Some authors hypothesized that it allows the cells to maintain a flexible gene expression program in order to be able to adapt more rapidly to changing environmental conditions^14,15^.

Recent work has shown that the frequency and length of transcriptional pulses could be actively regulated by external factors, for instance in the case of the negative coupling between oscillations in transcription of the *csaA* gene and extracellular cAMP levels in *Dictyostelium* or in that of the increased duration and frequency of pulsing of the mouse β-actin gene upon serum induction^16,17^. Of particular interest is the unresolved question of whether these parameters change during the cell cycle. Numerous studies have investigated gene expression during the cell cycle and subsets of genes that are periodically expressed at one point or another of the cell cycle have been readily identified^18–20^. However, most of these studies relied on measuring steady-state expression levels of cytoplasmic mRNAs in large cell populations, thus making it impossible to reach conclusions about nascent transcription at the single cell level.

Single molecule RNA FISH (smRNA FISH) is a powerful technique that enables the quantitative analysis of gene expression and nascent transcription at the single cell level^4,21^. Recently, Padovan-Merhar and colleagues used this technique to overcome previous methodological limitations and found that transcriptional output decreases on a per allele basis after DNA replication^22^. Skinner and colleagues confirmed these findings by performing simultaneous quantification of nascent and mature mRNA of *Pou5f1* and *Nanog*, well-known markers of pluripotency, in individual mouse embryonic stem cells^23^. Similar observations of dosage compensation after DNA replication were also made in yeast cells^24^.

Here we have used smRNA FISH, combined with live recording of cell growth, to study the dynamics of transcription during the cell cycle. In agreement with previous results^25^, we found that mitotic exit was accompanied by a transient spike in the transcriptional activity of the marker genes that were analyzed.

## Results

### smRNA FISH analysis of gene expression during the cell cycle

To image the dynamics of transcriptional pulsing throughout the cell cycle, we performed smRNA FISH on established human cell lines (HepG2, HT-1080 and U-2-OS) using fluorescent oligonucleotide probes against the transferrin receptor (TFRC) and the large subunit of RNA polymerase II (POLR2A). Both genes are ~30 kb in length, are moderately expressed in the chosen cell lines and are located in gene dense regions of the genome (HSA3q28 for TFRC and HSA17p13 for POLR2A). smRNA FISH dot-like signals were quantified using the MATLAB-based FISH-QUANT algorithm^26^. Based on DAPI staining, cells were classified as interphasic (G1/S/G2) or mitotic. For the latter, attention was given to cells in metaphase and in telophase. Cytometry of HepG2 cells immunostained for the serine 10-phosphorylated form of histone H3 revealed that ~3% were mitotic under the conditions used here. A cell was considered to be in telophase if it and one of its neighbors showed obvious signs of chromatin decondensation and mitotic exit, i.e. the shape of the DAPI signal was irregular, its intensity lied between that of metaphase and interphase cells, and cells had not yet flattened. Chromatin decondensation is triggered by the action of phosphatases such as PP2A-B55α during anaphase, as is cytokinesis^27,28^. Since both processes are concomitant and continuous, it is not always easy to distinguish between the end of telophase and the beginning of G1 based solely on DAPI staining and DIC images. For that reason, we refer to neighboring cells which display signs of chromatin decondensation as telophase/early G1 (telo/eG1).

**Figure 1:**
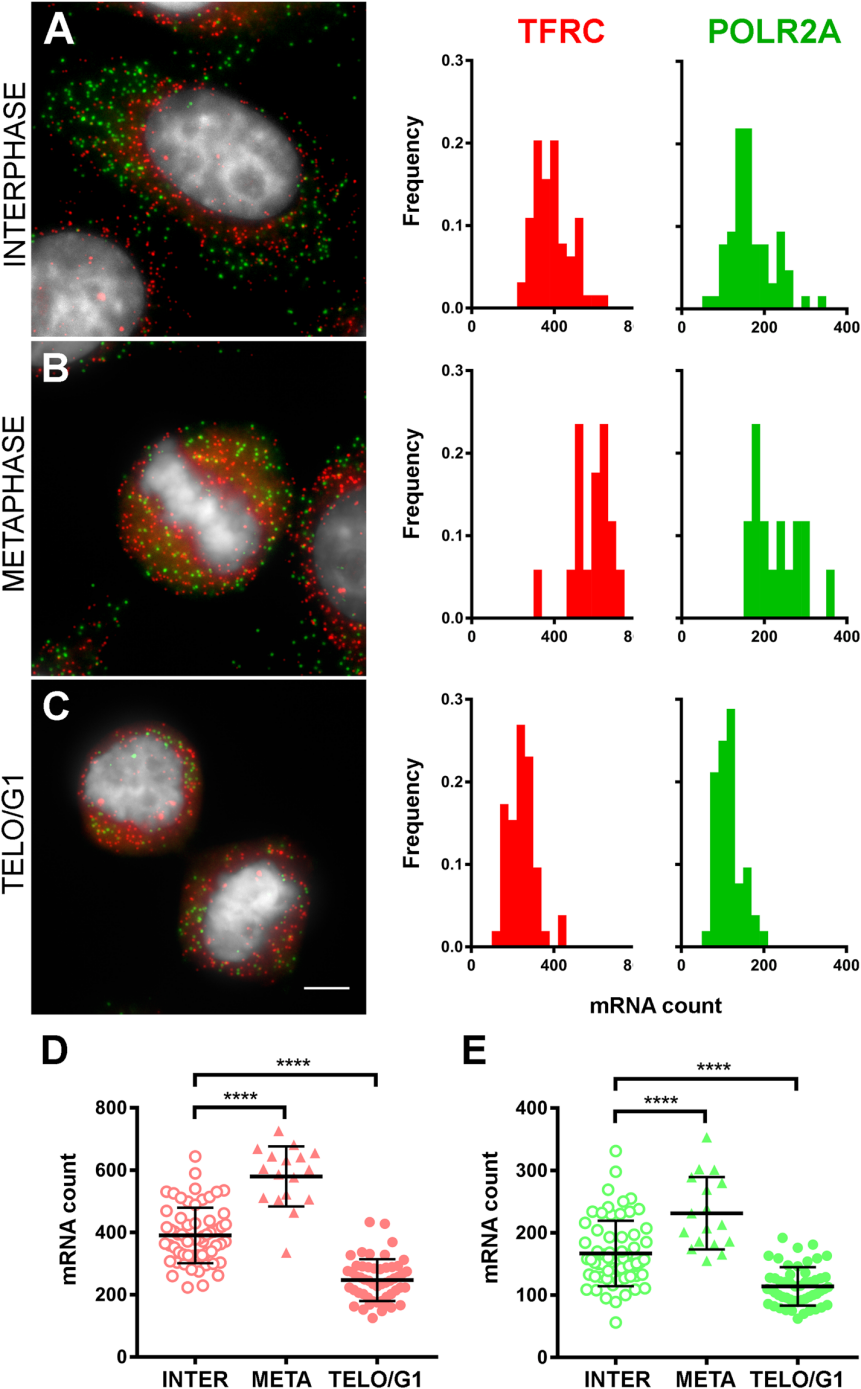
Quantitative smRNA FISH reveals gene expression dynamics in single cells during the cell cycle. (**A**–**C)** Representative smRNA FISH images of HepG2 cells are shown on the left for each of the target stages (**A**) interphase; (**B**) metaphase; (**C**) telophase/early G1). The images are projections of consecutive optical sections totaling 2 μm in thickness. Green dots, POLR2A RNA molecules; red dots, TFRC RNA molecules. DAPI counterstain in gray. Scale bar, 5 µm. Frequency distributions of mRNA counts are shown on the right (20 bins of equal size for each gene). **(D,E)** mRNA counts for TFRC (**D**, red) and POLR2A (**E**, green) at each target stage (n = 2 experiments; interphase, open circles, total of 64 cells; metaphase, triangles, total of 17 cells; telophase/early G1, filled circles, total of 52 cells). Each data point represents the mRNA count in an individual cell. Mean values (thick lines) ± standard deviation. ****p < 0.0001.

Figure 1 shows examples of smRNA FISH signals obtained for each of the 3 classes of HepG2 cells: interphase, metaphase, telo/eG1. All cells expressed TFRC and POLR2A. Quantification revealed a near-Gaussian distribution of cytoplasmic mRNA counts at all stages. The average mRNA counts were significantly different between the 3 stages (Fig. 1D,E). The mRNA count in metaphase cells was found to be ~1.5 fold higher than in interphase cells. As expected, the mRNA counts were halved in telo/eG1 cells when compared with metaphase cells (e.g. from 231 ± 58 to 114 ± 30 for POLR2A). Allowing for a smRNA FISH experimental error on the order of 10%, a clear majority of related daughter cells contained similar numbers of mRNA molecules (average ratio of 0.87 ± 0.09 for POLR2A and 0.89 ± 0.06 for TFRC, n = 50 daughter pairs). However, mRNA counts were found to differ by 20–40% in a significant fraction of daughter cell pairs, suggesting the possibility of biases in segregation of mRNA molecules during mammalian cell division (Supplementary Figure S1). These biases may contribute to gene expression noise at the population level, as previously suggested^29,30^. Results obtained in a different cell line (HT-1080) were similar to the ones described above for HepG2 cells (Supplementary Figure S2). Taken together, these quantitative smRNA FISH results shows an increase in mRNA counts during interphase and a halving in the number of mRNA after cell division.

### Transcription is increased in telophase/early G1 cells

**Figure 2:**
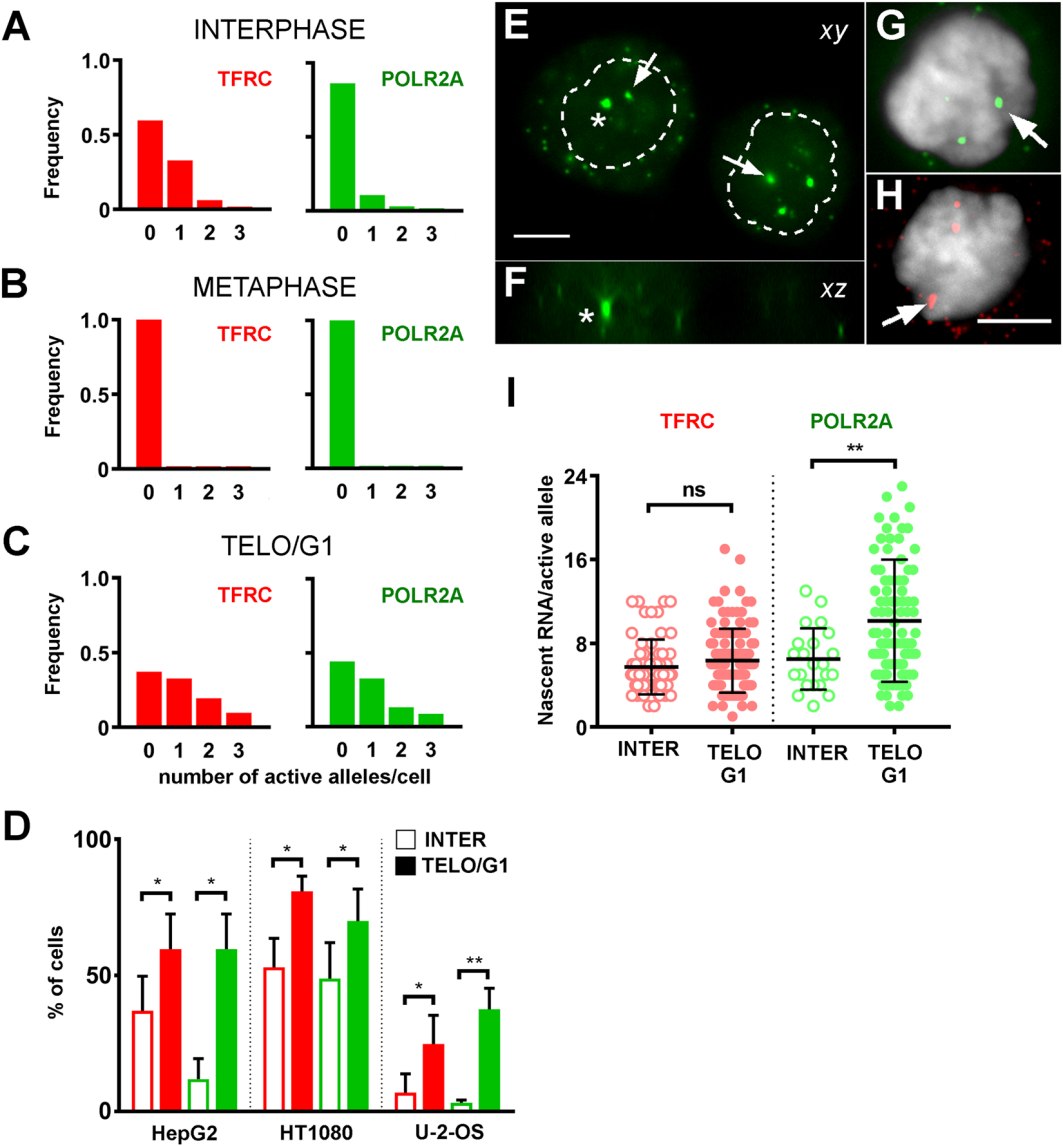
Transcription is increased upon mitotic exit. (**A–C)** Frequency distribution of the number of active alleles per HepG2 cell for TFRC (red) and POLR2A (green), at interphase (**A**, total of 131 cells), metaphase (**B**, total of 33 cells) or telophase/early G1 (**C**, total of 113 cells), n = 3 experiments. **(D)** Proportion of cells showing at least one active allele in interphase (open bars) or telophase/early G1 (filled bars). The data is shown for 3 different cell lines. Mean ± standard deviation of n = 3 experiments. *p < 0.05. **p < 0.01. **(E–H)** Representative images of smRNA FISH signals in a pair of daughter cells shortly after mitotic exit (**E**,**F**, POLR2A, green) or in individual nuclei (**G**, POLR2A, green; (**H**, TFRC, red). Shown are *xy* (**E**,**G**,**H**) projections of 2 consecutive optical sections (thickness of 0.5 μm). The *xz* projection (**F**) passes through one of the intense nuclear dots of the nucleus on the left in panel D (asterisks). Arrows point to some of the intense nuclear dots which mark putative transcription sites. Notice that the nuclear dots are clearly bigger than neighboring cytoplasmic dots and are often found in regions that weakly stain with DAPI. DAPI counterstain in gray. Contours of nuclei dotted. Scale bar, 5 µm. **(I)** Number of nascent RNA molecules per active allele in interphase cells (open circles) or in telophase/early G1 (filled circles). TFRC (red): interphase, total of 64 alleles; telophase/early G1, total of 117 alleles. POLR2A (green): interphase, total of 22 alleles; telophase/early G1, total of 100 alleles. n = 3 experiments. Mean values (thick lines) ± standard deviation. ns, not significant. **p < 0.01.

One of the advantages of imaging RNA at the single cell level is the ability it affords to distinguish between mature cytoplasmic mRNA molecules and nascent transcripts. Indeed, because of rate-limiting steps in pre-mRNA processing, nascent transcripts are detected as intense dots in the nucleus^4^. When the frequency of such dots was determined for TFRC and POLR2A in HepG2 populations, the majority of cells were found not to have been transcribing the genes at the time of fixation (Fig. 2A). For POLR2A, an average of 87% of cells did not display any nuclear dots (n = 3 experiments, total of 131 cells). This is consistent with both genes being transcribed in a pulsatile manner, with relatively short periods of activity interspersed with periods of inactivity. Consistent with the global shutdown of transcription that is observed during mitosis^31^, no nuclear dots were found in metaphase cells (Fig. 2B). The most interesting observation about nascent transcription was made on telo/eG1 cells (Fig. 2C), in which a marked increase in the number of nuclear dots was observed, both in terms of the proportion of positive cells (63% for TFRC and 56% for POLR2A compared to 43% and 13%, respectively, in interphase cells) and with regard to the average number of active alleles per cell (1.04 ± 0.10 for TFRC and 0.89 ± 0.09 for POLR2A compared with 0.50 ± 0.06 and 0.17 ± 0.04, respectively, in interphase cells). In a significant proportion of cells, 3 alleles were found to be simultaneously transcribed in telo/eG1 cells, a situation that was very rarely observed in interphase cells. Karyotype and FISH analysis confirmed the suspected aneuploidy of the HepG2 cells that were used and the associated polysomy of the target genes (Supplementary Figure S3). The increase in the proportion of cells displaying active alleles at telo/eG1 was observed in all three cell lines that were analyzed (Fig. 2D). Representative *xy* and *xz* projections of the POLR2A signal in telo/eG1 cells are shown on Fig. 2E,F. Note that the nuclear dots which correspond to accumulation of nascent transcripts are many times bigger than the cytoplasmic dots, which correspond to single mature mRNA. Results obtained on HT-1080 cells were similar to the ones described here for HepG2 cells (Supplementary Figure S4).

Upon closer inspection of individual optical sections, the intense nuclear dots were often found to be localized to regions of low DAPI intensity (Fig. 2G,H), pointing to a link between chromatin decondensation and resumption of transcription. This observation was quantified by placing the DAPI intensity measured at the transcription spot on a distribution of binned DAPI pixel values in a region-of-interest of ~3 μm × 3 μm around the transcription spot. This analysis was performed for 15 transcription spots for each gene and showed that these were found in regions of low local DAPI intensity in 12/15 cases for POLR2A and 15/15 cases for TFRC (Supplementary Figure S5).

The number of RNA molecules within the nascent transcription dots can be estimated through comparison with the signals produced by single mRNAs in the cytoplasm. The transcriptional output per active allele was found to be similar between interphase and telo/eG1 cells in the case of TFRC (5.7 ± 2.6 vs. 6.5 ± 2.9 nascent RNA molecules/active allele, Fig. 2I, red), but significantly increased in the case of POLR2A (6.3 ± 3.0 vs. 10.2 ± 5.8 nascent RNA molecules/active allele, Fig. 2I, green). This observation indicates that both the transcriptional burst fraction and burst size can be increased upon mitotic exit. Taken together, our results suggest that exit from mitosis is accompanied by a marked surge in nascent transcription.

**Figure 3:**
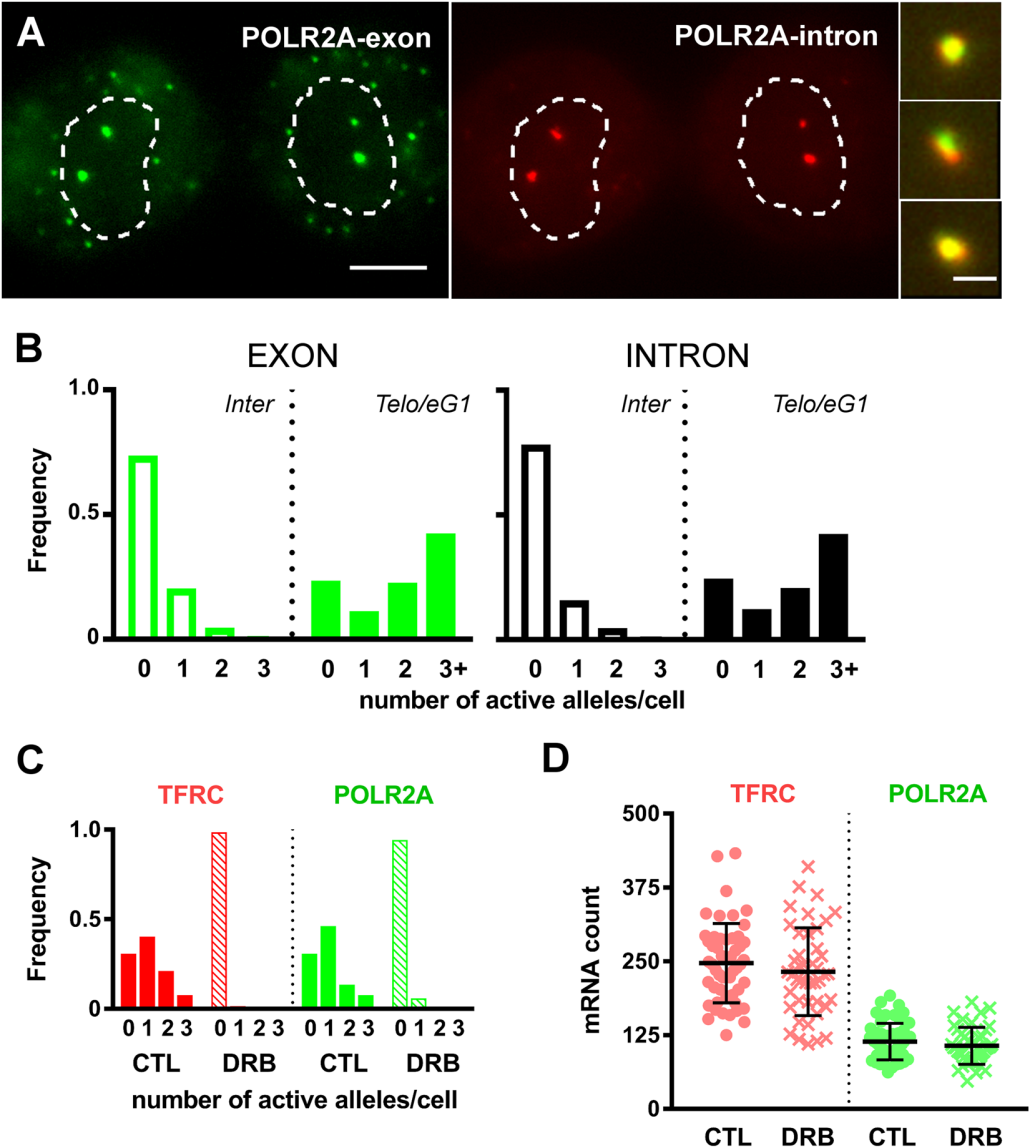
Intense nuclear smRNA FISH signals mark sites of nascent transcription. **(A)** Representative smRNA FISH images of HepG2 telophase﻿/early G1cells hybridized simultaneously with an exonic probe against POLR2A (left panel, green) and a probe against the first intron of POLR2A (middle panel, red). Contours of nuclei are dotted. Scale bar, 5 µm. Examples of enlarged transcriptional spots showing co-localization of the exon and intron signals in the nucleus are on the right. Scale bar, 1 μm). All images are projections of two consecutive optical sections totaling 0.5 μm in thickness. **(B)** Frequency distributions of the number of active POLR2A alleles per cell in interphase (open bars, 152 cells) and telophase/early G1 (filled bars, 106 cells) detected using the exon (left) or intron (right) probes. **(C)** Cells were treated with an inhibitor of transcriptional elongation (DRB) for 1 hour before being processed for smRNA FISH. Frequency distribution of the number of intense nuclear dots per HepG2 cell in telophase/early G1 for TFRC (red) and POLR2A (green) in control (CTL) cells (filled bars, a total of 52 cells) or DRB-treated cells (dashed bars, a total of 44 cells). n = 2. (**D)** mRNA counts for TFRC (red) and POLR2A (green) in telophase/early G1 cells that were either treated with DRB (crosses) or not (filled circles). Mean values (thick lines) ± standard deviation.

The identity of the intense smRNA FISH dot-like signals in the nucleus with nascent transcripts at the site of synthesis was ascertained in the following ways. First, in the case of POLR2A, intense nuclear dots that were detected using the exonic probe were also labeled with a probe against the first intron of the gene (Fig. 3A). This result indicates that these signals arise from *bona fide* pre-mRNA molecules and not from random entrapment of processed transcripts during chromatin decondensation. Signals from exonic and intronic probes were co-localized in 99% of cases (209 out of 212 signals analyzed in telo/eG1 nuclei). Accordingly, the observation of transcriptional spiking upon mitotic exit was duplicated using the intron probe (Fig. 3B). Second, treatment of cells with 5,6-dichlorobenzimidazole 1-β-D-ribofuranoside (DRB), a CDK9 kinase inhibitor that blocks transcriptional elongation^32^, for 1 hour before fixation led to the complete disappearance of nuclear dots from telo/eG1 cells (Fig. 3C). The cytoplasmic mRNA counts were not significantly affected by this brief treatment (Fig. 3D), consistent with the reported half-lives of 5 and 10 hours for POLR2A and TFRC in HepG2 cells, respectively^33^. The disappearance of nuclear dots was also observed with two other transcriptional inhibitors, flavopiridol and actinomycin D (Supplementary Figure S6). Taken together, these results confirm that the intense smRNA FISH signals found in the nuclei label accumulation of nascent transcripts at the site of synthesis. These will now be referred to as transcriptional spots.

### Post-mitotic increases in transcriptional output are transient

**Figure 4:**
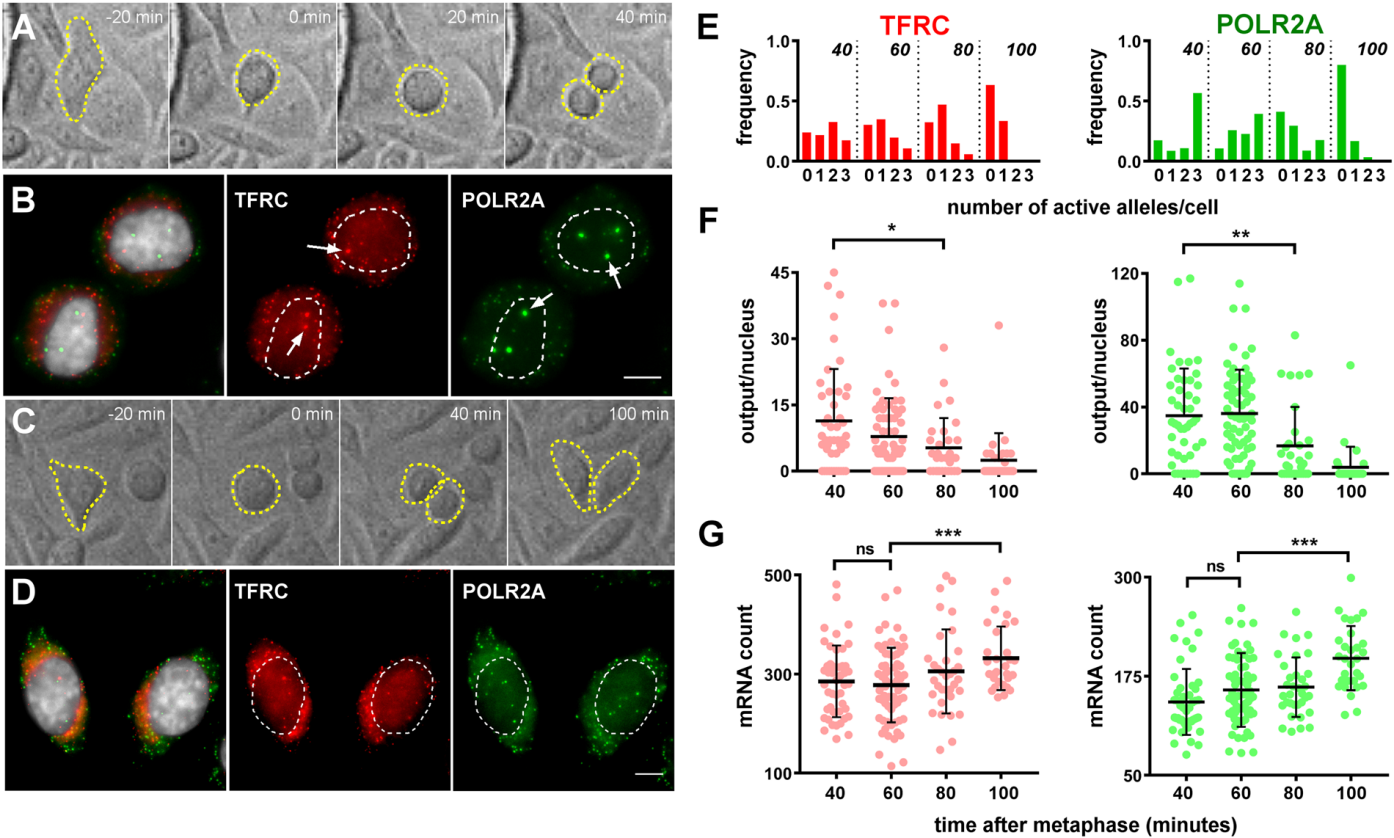
Post-mitotic transcriptional spikes occur early and are transient. (**A,C)** Snapshots taken from a movie of HepG2 cell growth. When recording was stopped (rightmost panels), the pair of daughter cells in A was fixed 40 minutes after metaphase had occurred (second panels) while the pair in C was fixed 100 minutes after metaphase. **(B,D)** smRNA FISH images for the pair of daughter cells shown in A and C, respectively. The TFRC signal (middle panels) is pseudo-colored in red, the POLR2A signal (right panels), in green. The left panels show a merged image with DAPI counterstain in gray. Arrows in B point to transcription spots. Maximum intensity projections of 4 consecutive optical sections (thickness of 1 μm). Scale bars, 5 μm. **(E**–**G)** Quantification of smRNA FISH signals for TFRC (red) and POLR2A (green) in HepG2 cells at different times after metaphase (n = 3. t = 40 minutes, 46 cells; t = 60 minutes, 66 cells; t = 80 minutes, 34 cells; t = 100 minutes, 30 cells). Shown are frequency distribution of the number of active alleles per nucleus (**E**), the cumulative output per nucleus (F) and the cytoplasmic mRNA count (**G**). Mean values (thick lines) and standard deviations are indicated on scatter dot plots (**F**,**G**). ns, not significant. *p < 0.05. **p < 0.01. ***p < 0.001.

The use of unsynchronized fixed cells made it difficult to precisely define the time window during which cells display spikes of nascent transcription. To circumvent the limitation inherent to working on fixed material, we chose to record the growth of cells in real time before performing smRNA FISH. Cells were seeded on gridded glass coverslips and immediately fixed and processed at the end of the recording period (7–17 hours at 20-minutes intervals). Movies were scanned to identify daughter cells at different time points after mitosis. After hybridization, these cells were localized on the mounted gridded coverslip and imaged. The time point at which a metaphase plate was observed was taken as t = 0. Snapshots from representative movies, along with the smRNA FISH results at endpoint, are shown on Fig. 4A–D. At t = 20 minutes, cells were still in anaphase. Multiple transcriptional spots were already seen at the next time point (t = 40 minutes), when cytokinesis appeared to be almost completed but cells were still rounded. One hour later (t = 100 minutes), the number of transcriptional spots had gone down to the levels that were observed in populations of interphase cells, with few instances of nuclei displaying more than one active allele. The frequency distributions of the number of active alleles per nucleus (0, 1, 2 or 3) was progressively shifted to the left as cells progressed from telophase to G1 (Fig. 4E). Accordingly, quantification of signals at the different time points revealed a progressive decrease in the transcriptional output per nucleus, calculated as the sum of the number of nascent transcripts found at each allele (Fig. 4F). For POLR2A for instance, the mean transcriptional output per nucleus went from 34 ± 4 nascent transcripts 40 minutes after metaphase (n = 3, 46 cells) down to 4 ± 2 100 minutes after metaphase (n = 3, 30 cells). Counting the number of mature mRNA molecules in the same cells revealed that the elevated transcriptional output at early time points was followed by a statistically significant (p < 0.001) increase in mRNA counts for both genes at the last time point (Fig. 4G; +19% in the case of TFRC and +25% for POLR2A). Interestingly, these increases in cytoplasmic mRNA counts were found to be more or less equivalent to the cumulative number of nascent transcripts detected at earlier time points, suggesting that the occurrence of transcriptional spikes upon mitotic exit might serve to rapidly increase the number of mature mRNAs in daughter cells. Since the kinetics of transcription, RNA processing and RNA export remain largely unknown in the reforming nucleus, additional work in single living cells is needed to assess the exact contribution of the post-mitotic spikes that we observe to the cytoplasmic mRNA pools.

Taken together, these results show that the increase in transcription in telo/eG1 cells is 1) maximal during chromatin decondensation, upon completion of cytokinesis but before the flattening of daughter cells; and 2) transient, at most lasting less than one hour. The early and transient nature of the transcriptional spike we observed was further revealed by co-staining for the abscission midbody, a β-tubulin-rich structure that persists up to 3 hours after mitotic exit^34^. Analysis of immuno-RNA FISH results showed that only a subset of cells still linked by a midbody, and hence at the beginning of G1, displayed intense transcriptional spots in the nucleus (Supplementary Figure S7).

### Distinct regulation of transcriptional spikes

**Figure 5:**
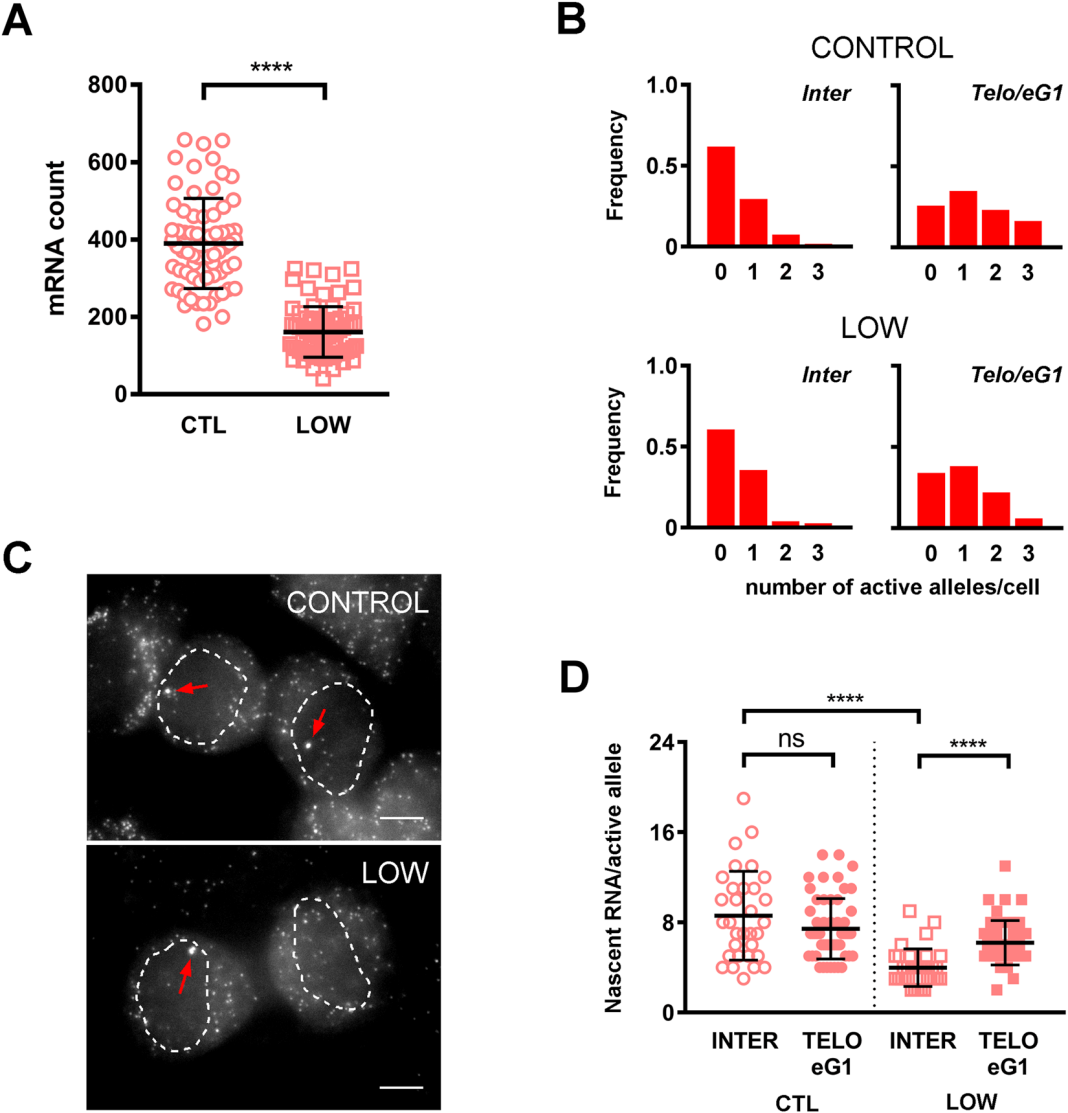
Distinct transcriptional regulation in telophase/early G1 HepG2 cells. (**A)** Interphase mRNA counts for TFRC in cells cultivated in medium supplemented with 10% (CTL, open circles, n = 2 experiments, total of 68 cells) or 0.2% (LOW, open squares, n = 2 experiments, total of 76 cells) fetal bovine serum. Each data point represents the mRNA count in an individual cell. Mean values (thick lines) ± standard deviation. ****p < 0.0001. **(B)** Frequency distribution of the number of active TFRC alleles per nucleus in interphase (left) and telophase/early G1 (right) for cells grown in normal medium (top) or low-serum medium (bottom). The combined results from 2 experiments and 3 biological replicates are plotted. The total numbers of cells included in each analysis are: 68 for control interphase, 76 for low-serum interphase, 43 for control telophase/early G1 and 50 for low-serum telophase/early G1. **(C)** Representative smRNA FISH images for TFRC in telophase/early G1 cells grown in normal (top) or low-serum (bottom) conditions. In both cases, transcriptional spots of similar intensity are detected (arrows). For clarity, the images are maximum intensity projections of only 4 consecutive optical sections totaling 1 μm in thickness. The contours of the daughter cell nuclei are dotted. Scale bar, 5 µm. **(D)** Number of nascent RNA transcripts per active allele in interphase cells (INTER, open symbols) or telophase/early G1 cells (TELO/eG1, filled symbols). In control conditions (CTL, circles), the mean allele activity is the same at the two stages (INTER, 32 alleles; TELO/eG1, 56 alleles). In low serum conditions, the mean allele activity is significantly increased upon mitotic exit (INTER, 33 alleles; TELO/eG1, 49 alleles). Mean values (thick lines) ± standard deviation. ns, not significant. ****p < 0.0001.

The transcriptional dynamics of the genes that were analyzed were found to be different in telophase/early G1 and in interphase cells. It was therefore of interest to determine whether gene regulation occurred through similar mechanisms at these two stages of the cell cycle. In other words, we wanted to address the issue of whether the transcriptional burst fractions and burst sizes during interphase somehow determined those of the post-mitotic transcriptional spikes. To do so, we took advantage of the fact that the TFRC gene is known to be upregulated by mitogenic stimuli^35,36^. Hence, cultivating cells in low serum is a simple and efficient mean of lowering transcription of this gene. As expected, incubation of HepG2 cells in medium supplemented with 0.2% serum for 48 hours led to a 2.4-fold decrease in average mRNA counts in interphase cells (Fig. 5A). This decrease was not a consequence of changes in burst fraction, as the proportions of active alleles per cell were comparable for both culture conditions (Fig. 5B). Rather, the decrease in TFRC mRNA counts in low serum was a result of decreased output per active allele (8.6 ± 4.0 vs. 4.0 ± 1.7 nascent RNA molecules/active allele, Fig. 5D). When the analysis was focused on transcriptional spots in telo/eG1 cells however, we found only a slight reduction in allelic output in low serum conditions (7.4 ± 2.7 vs. 6.2 ± 2.0 nascent RNA molecules/active allele, Fig. 5D). Unlike what was observed for interphase cells, the majority of transcriptional spots were of similar intensities in telo/eG1 cells irrespective of whether these were grown in normal or low serum conditions (Fig. 5C). Taken together, these results suggest that distinct regulatory mechanisms may be at work shorty after mitotic exit and during the rest of interphase.

## Discussion

Gene regulation during the cell cycle has been intensively studied. Because of the ease with which they can be synchronized and the availability of a large collection of cell cycle mutants, many studies have used yeast cells to determine gene expression levels at different stages of the cell cycle. Initial studies revealed a stepwise doubling in the overall rate of RNA synthesis shortly after DNA replication^37^. Recent work pointed out that this difference in transcriptional activity between G1 and S/G2 was largely responsible for the cell-to-cell variability in expression levels in populations of *S. cerevisiae* cells^38^. In mammalian cells, the long G1 phase, the variability in cell cycle length and the buffering effects of long mRNA half-lives complicate the analysis of causal relationships between the cell cycle and transcriptional output. Recently, Skinner *et al*. quantified expression of two pluripotency-associated genes across the cell cycle in single embryonic stem cells and found a difference of 1.6-fold between mRNA counts in the G1 and G2 phases^23^. We measured a similar difference when comparing mRNA counts of TFRC and POLR2A in interphase and metaphase cells.

Direct measurement of the proportion of active alleles and of the number of nascent RNA molecules affords a powerful mean to investigate transcriptional dynamics. In their study, Skinner *et al*. measured these parameters and found evidence for dosage compensation, i.e. a decrease in bursting fraction, after DNA replication^23^. Dosage compensation after DNA replication was also observed in budding yeast, where it was found to depend on acetylation of K56 on newly deposited histones H3^24^.

In the present study, quantification of nascent transcription sites led to the realization that transcriptional output was maximal upon mitotic exit. Previous work hinted in one way or another at this possibility. For instance, microarray analysis of nascent RNA labeled at different times after release from nocodazole arrest identified several genes whose expression peaked in early G1. Furthermore, based on the live imaging of the activity of the *act-5* genes in *Dictyostelium*, Muramoto and colleagues reported an increase in transcriptional activity shortly after mitosis^19,39^. However, for the most part these previous observations were not interpreted as transcriptional spikes. On the other hand, a recent whole-genome ChIP analysis revealed a spike in RNA polymerase II occupancy at ~50% of bound genes in early G1 murine erythroblast cells^25^. smRNA FISH was used to confirm the existence of transcriptional spikes for the *Myc* and *Gata2* genes upon mitotic exit. While our results are in general agreement with this previous report, they differ in two important respects. First, the authors of this study found that the transcriptional spikes in telophase/early G1 arose solely from an increase in the proportion of active alleles (burst fraction), with unchanged output per active allele (burst size). Whereas in our study the TFRC gene conforms to this interpretation, we did detect an increased number of nascent transcripts per active allele in the case of the POLR2A gene, suggesting that the post-mitotic activity of different genes can spike through different mechanisms. In interphase cells, gene-specific pulsing parameters have been reported for developmental and housekeeping genes in *Dictyostelium* ^40^. Similarly, serum regulation of the β*-actin* gene has been shown to proceed through modulation of both frequency and size of transcriptional pulses^17^. The use of engineered transcriptional activators revealed that the pulse frequency of the *c-fos* gene was linked to the strength of the transactivation domain whereas the pulse size correlated with the binding affinity of the activator to the gene promoter^41^. In the two-state model of transcriptional pulsing, burst fraction and burst size are associated with the rate of gene switching to a transcriptionally-permissive state and with the rate of transcriptional initiation, respectively^42^. A more recent model proposes that bursting is characterized by a continuum of transcriptional states, emphasizing that the transcriptional initiation rate is unlikely to be constant over time even during a single burst^43^. Consistent with this model, we show here that initiation rates are not constant during the cell cycle. The second difference between our results and those of Hsiung *et al*. is the following. These authors noted that the transcriptional spikes occurred at a time when chromosomes are still morphologically condensed. We have made a similar observation. However, closer examination of the image sections revealed that the vast majority of transcriptional spots were found in regions of low DAPI intensity, which leads us to speculate on a possible link between post-mitotic transcriptional spikes and chromatin decondensation. Such a link would be consistent with our recent results that showed that chromatin decondensation induced by changes in extracellular osmolarity in living interphase cells was accompanied by a transient increase in the transcriptional output of a tagged transgene^44^.

Cell division entails a transient but profound reorganization of nuclear architecture. The condensation of chromatin is particularly obvious during mitosis. *In situ* imaging revealed that chromatin subdomains are linearly compressed along the mitotic chromosomes^45^. Conformational analysis using the Hi-C techniques confirmed these observations and further showed that the organization of chromatin into self-interacting modules referred to as topologically-associated domains, or TADs, is lost in metaphase chromosomes^46^. The reorganization of chromatin during telophase/early G1 has been much less studied than during entry into mitosis. It can nonetheless be assumed that chromatin decondensation proceeds in steps that are the reverse of those which lead to chromatin condensation, i.e. through de-compression of chromosomes and reformation of TADs. According to a current model, the existence of TADs is the result of dynamic chromatin loop extrusion^47,48^. Hence, we suggest that the massive re-structuring of TADs throughout the chromatin fiber upon mitotic exit is accompanied by highly dynamic chromatin looping. Interestingly, chromatin looping through dynamic contacts between enhancer and promoter regions has recently been shown to be linked to transcriptional bursting^49,50^.

Upon mitotic exit, the rapid decondensation of chromatin could lead to local entrapment of the components of the transcriptional machinery, which are known to have entered back into the nascent nucleus at this time^51^. This entrapment could in turn lead to increases in the local concentration of RNA polymerase II and hence to increased initiation rates and thus transcriptional spikes. This hypothesis is consistent with previous observations of an accumulation of an inert marker in the chromatin in late mitosis^52^. A highly dynamic nature of chromatin states could also help to explain our important finding that the regulation of transcription in telophase/early G1 appears to be distinct from regulation later in interphase, as shown by the maintenance of high intensity post-mitotic transcriptional spikes of the TFRC gene despite cells being cultured in conditions which lead to a 2.5 fold decrease in the intensity of transcriptional spots during interphase. Additional work is needed to identify the mechanisms that are responsible for the increase that we have observed in the rate of transcriptional initiation at the beginning of the cell cycle.

What could be the biological significance of post-mitotic transcriptional spikes, if any? They can be viewed as a compensatory response to the halving of mRNA counts during cell division. We have indeed observed a step increase in mRNA counts shortly after the birth of daughter cells. It has been proposed that unequal spike intensities in the two daughter cells could lead to increased cell-to-cell variability in gene expression across the cell population^25^. As mentioned above, we favor the hypothesis that transcriptional spikes are causally and mechanistically linked to the remodeling of chromatin that occurs after mitotis. As shown here for the TFRC gene, we hypothesize that chromatin decondensation is conducive to a dysregulation of gene activity that might affect a large part of the genome. This hypothesis is consistent with recent work that showed that mitosis provides a window of opportunity for reprogramming or that certain changes in cell fate preferentially occur during the early G1 phase of the cell cycle^53,54^.

## Methods

### Cell culture, drug treatment and reagents

HepG2 (ATCC no. HB-8065) and HT-1080 (ATCC no. CCL-121) cells were routinely cultured in DMEM containing 4.5 g/l of glucose and supplemented with 10% fetal bovine serum (FBS), penicillin (50 units/ml) and streptomycin (50 μg/ml). U-2-OS (ATCC no. HTB-96) cells were cultured in DMEM containing 1.5 g/l glucose and the same supplements. For treatments with 5,6-dichlorobenzimidazole 1-β-D-ribofuranoside (DRB, 100 μM), flavopiridol (1 μM) and actinomycin D (1.5 μg/ml), cells were incubated in medium containing the drug at the indicated concentration for 1 hour before being processed for smRNA FISH. For culture in low serum conditions, HepG2 cells were extensively washed with Hanks’ buffered saline solution and incubated for 2 days in low glucose DMEM supplemented with 0.2% FBS. Cell culture reagents were purchased from ThermoFisher/Life Technologies. Chemicals were from Sigma-Aldrich unless stated otherwise.

### Single molecule RNA FISH

The smRNA FISH protocol is based on previously published ones^4^. Briefly, cells were grown on uncoated 18 mm × 18 mm #1.5 coverslips, fixed with 4% formaldehyde in 1X PBS for 10 minutes, washed in 1X PBS and stored overnight in 70% ethanol at 4 °C. After rehydration, hybridization with probes at final concentrations of 62 nM was carried out in 2X SSC/10% formamide/10% dextran sulfate for 6–14 hours at 37 °C. The hybridization mix also contained 10 mM ribonucleoside vanadyl complex (New England Biolabs) and in some experiments 100–200 μg/ml of yeast tRNA to protect cellular RNAs. After washing with 10% formamide/2X SSC for 30 minutes at 37 °C and twice with 2X SSC for 5 minutes at room temperature, samples were stained with 4′,6-diamidino-2-phenylindole (DAPI, 1 μg/ml in 2X SSC) and mounted in Prolong Gold (ThermoFisher/Life Technologies). Slides were cured at room temperature for 2–3 days before imaging. Oligonucleotide (20-mer) probes against human POLR2A (n = 48, Supplementary Table S1), the first intron of human POLR2A (n = 47, Supplementary Table S2) and human TFRC labeled at the 3′ end with Quasar 670 (λ_ex_ = 646 nm, λ_em_ = 670 nm), Quasar 570 (λ_ex_ = 548, λ_em_ = 566) and Quasar 570 dyes, respectively, were purchased from BioSearch Technologies. All experiments were performed at least in duplicates.

### smRNA FISH image acquisition and analysis

Optical sections (30–40 at 250 nm intervals) were acquired on an Olympus IX71 inverted epifluorescence microscope using a 100X/NA1.3 oil immersion objective, an Andor Clara CCD camera (Andor Technology, Belfast, UK) and an automated piezo-Z stage (Prior Scientific Instruments, Cambridge, UK). Typical *xy* pixel size was 67 nm. The dynamic range of the images was 12 bits. The counting of cytoplasmic mRNAs as well as the quantification of the nascent transcription signals were peformed using the MATLAB-based FISH-QUANT program (https://code.google.com/p/fish-quant/)^26^. Cells were segmented manually. Typically, images were filtered using Gaussian kernels of 5 (for background subtraction) and 0.5 (for feature enhancement). For mRNA counts, images were processed in batches and randomly validated. All transcriptional spots included in our study were identified by virtue of being brighter than single cytoplasmic molecules, either manually or using the thresholding function of the FISH-quant software. All transcriptional spots were validated visually. The number of RNA molecules that make up an individual transcription site was determined using the point-spread-function (PSF) superposition approach in FISH-QUANT. Since we found that they showed the same trend, we chose to pool quantification results from independent smRNA FISH experiments.

### Live recording of cell growth

Cells (1 to 2 × 10^5^) were seeded in glass bottom, gridded 35mm petri dish (Ibidi, cat. no. 81168) and left to grow to ~50% confluence. One hour before imaging, the medium was changed to CO_2_-independent medium (ThermoFisher/Life Technologies) supplemented with serum and antibiotics. Growth was recorded with a 20X oil objective on a Leica SP5 confocal microscope. Optical stacks (z-step of 3 μm, image size of 512 pixels ×512 pixels) were recorded for 25 positions at 20-minute intervals. The duration of movies ranged from 7–17 hours. Once recording was stopped, cells were immediately fixed. smRNA FISH was performed as described above, except that hybridization was carried out directly in the petri dish as follows. A 100 μl drop of hybridization mix was pipetted on a round 12 mm coverslip, which was then flipped onto the central part of the gridded coverslip. A 22 mm × 22 mm coverslip was put on top of the hybridization sandwich to prevent drying. After washes, cells were mounted with Prolong Gold. Cells fixed at different times after division were identified from the movies and their positions determined on the grid, which allowed to trace them back on the mounted coverslips and thus image smRNA FISH signals at specific time points of the cell cycle.

### Data Availability

The datasets generated during and/or analysed during the current study are available from the corresponding author on reasonable request.

## Electronic supplementary material

Supplementary Info
