# Supplementary material for: Transcriptional Output Transiently Spikes Upon Mitotic Exit
Source: Sci Rep. 2017 Oct 3;7:12607. doi: 10.1038/s41598-017-12723-7 (PMC5626720; doi:10.1038/s41598-017-12723-7)
Supplement: Supplementary file 1 — Supplementary Info [file 41598_2017_12723_MOESM1_ESM.pdf]

SUPPLEMENTARY INFORMATION

Transcriptional output transiently spikes upon mitotic exit

Viola Vaňková Hausnerová[1, 2], Christian Lanctôt[1*]

[1]BIOCEV and Department of Cell Biology, Faculty of Science, Charles University, Prague, Czech Republic,

[2]First Faculty of Medicine, Charles University, Prague, Czech Republic

Supplementary Methods

Preparation of metaphase spreads

Growing cells (HepG2 and HT-1080) were incubated in medium containing 0.1 μg/ml colchicine for 20 minutes. Cells were then trypsinized, incubated in an hypotonic solution (0.56M KCl) for 12 minutes at 37ºC, and fixed with methanol:acetic acid (3:1) for 20 minutes at -20ºC. After several washes, fixed cells were dropped on microscope slides in a humidified chamber in a water bath at 55ºC and left to dry for 2 minutes. Metaphase spreads were aged at room temperature for at least 5 days.

FISH on metaphase spreads with BAC probes

Bacterial artificial chromosomes (BAC) comprising the human POLR2A gene (RP11-104H15, CHORI BACPAC Resource Center) or sequences 4 Mb centromeric to the human TFRC gene (3q28, RP11-183L23, a kind gift of Dr. Marion Cremer, Ludwig-Maximilians Universität München) were extracted from 1 ml of bacterial cultures and linearly amplified using the GenomiPhi V2 kit according to the manufacturer's instructions (GE Healthcare Life Sciences). Labeling of BAC DNA and FISH on metaphase spreads were performed as previously described [1]. Briefly, DNA was labeled either with DIG (POLR2A) or biotin (3q28). Before hybridization, samples were treated with pepsin at 100 μg/ml in 0.01N for 8-9 minutes at room temperature, dehydrated and air-dried. Probes (at final concentration of 5 ng/μl) and target DNA were denatured simultaneously by placing the slides on a hot block at 76ºC for 2 minutes. Hybridization was carried out at 37ºC for 2 days. After washes, hybridized probes were detected with antibodies against DIG coupled to Cy5 (POLR2A) or streptavidin coupled to Cy3 (3q28/TFRC). Samples were counterstained with DAPI and imaged on a Nikon TiE widefield fluorescence microscope.

Analysis of the mean DAPI intensity at transcriptional spots

We have quantified the DAPI signal intensity in the regions of transcriptional spots in the telophase/early G1 nuclei in the following way. 1) the *z*-section containing the focus of a given transcription spot was determined manually; 2) $DAPI^{spot}$, the pixel value at the spot maxima was determined in the corresponding DAPI channel; 3) the DAPI pixel intensity values were determined in a region-of-interest of ~3 μm x 3 μm (50 pixels by 50 pixels) drawn around the transcriptional spot and distributed into 256 bins of equal size; 4) the bin comprising the $DAPI^{spot}$ value was noted. The above steps were repeated for 15 spots for each gene. Results are plotted separately for individual spots.

Immuno-RNA FISH to detect tubulin along with TFRC and POLR2A RNAs

HepG2 cells were grown on uncoated 18 mm x 18 mm #1.5 coverslips, fixed with 4% formaldehyde in 1X PBS for 10 minutes, washed and stored overnight in 70% EtOH at 4°C. After rehydration in PBS, cells were blocked for 7 minutes in PBS containing 1 mg/ml bovine serum albumin and 10mM ribonucleoside vanadyl complex (New England Biolabs), which was also included in all antibody solutions during the subsequent immunostaining procedure. Cells were then incubated in a 1:20 dilution (in PBS) of a mouse monoclonal antibody against β-tubulin (clone E7, Developmental Studies Hybridoma Bank at the University of Iowa) for 30 minutes at room temperature. After brief washes with PBS (2 times, 2 minutes each), cells were incubated in a 1:200 dilution (in PBS, final concentration of 6.5 μg/ml) of a secondary biotinylated goat anti-mouse antibody (Jackson Labs) for 30 minutes at room temperature. After brief washes with PBS (3 times, 2 minutes each), the immunocomplexes were fixed for 10 minutes with 2% (v/v) formaldehyde in PBS. After successive washes with PBS (1 minute) and 2X SSC (2 minutes), followed by equilibration in 2XSSC/10% formamide (5 minutes), smRNA FISH was performed as described in Materials and Methods. After the last

wash with 2X SSC, cells were further stained for 20 minutes at room temperature.with DAPI (1 μg/ml) and a 1:400 dilution of avidin-Alexa 488 (final concentration of 2.5 μg/ml, ThermoFisher/Life Technologies). Finally, samples were washed twice with 2X SSC and mounted in Prolong Gold.

References for supplementary methods

1 Cremer, M. *et al.* Multicolor 3D Fluorescence In Situ Hybridization for Imaging Interphase Chromosomes. *Methods Mol Biol* **463**, 205-239 (2008).

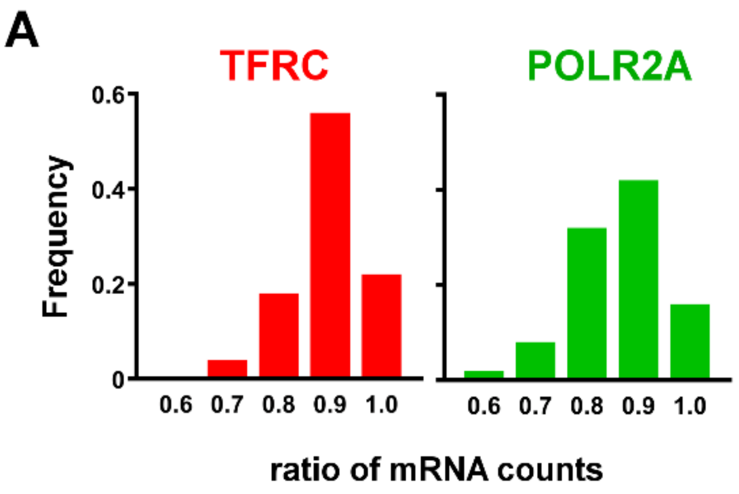

Figure S1. Segregation of mRNA molecules during cell division. (A) Frequency distributions of the ratios of mRNA counts in pairs of HepG2 daughter cells, in bins of 10%, for TFRC (red) and POLR2A (green). The combined results from 3 independent experiments are plotted (total of 50 pairs of daughter cells). Allowing for a smRNA FISH experimental error on the order of 10%, a clear majority of related daughter cells contain similar numbers of mRNA molecules (ratios of 0.9-1). However, mRNA counts can differ by up to 40% in a significant fraction of daughter cell pairs (ratios 0.6-0.8).

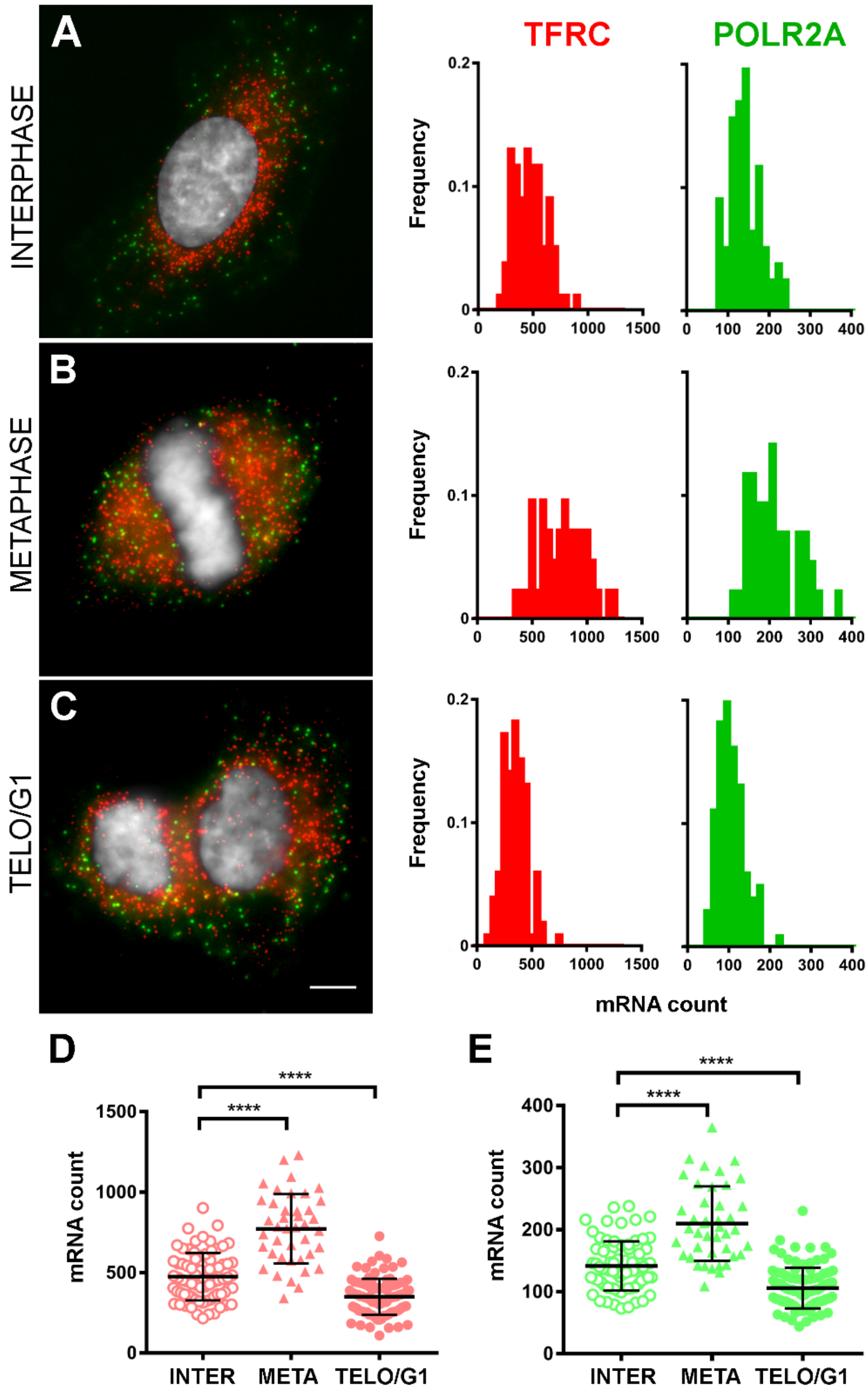

Figure S2. Quantitative smRNA FISH results for HT-1080 cells. (A-C) Representative smRNA FISH images of HT-1080 cells are shown on the left for each of the target stages (A, interphase; B, metaphase; C, telophase/early G1). The images are projections of consecutive optical sections totaling 2 μm in thickness. Green dots, POLR2A RNA molecules; red dots, TFRC RNA molecules. DAPI counterstain in gray. Scale bar, 5 μm. Frequency distributions

of mRNA counts in the population of cells that were analyzed are shown on the right (27 or 26 bins of equal size for each gene). **(D-E)** mRNA counts for TFRC (D, red) and POLR2A (E, green) at each target stage (n = 2 experiments: interphase, open circles, total of 76 cells; metaphase, triangles, total of 42 cells; telophase/early G1, filled circles, total of 98 cells). Each symbol represents the mRNA count in an individual cell. Mean values (thick lines) ± standard deviation. ****, $p < 0.0001$.

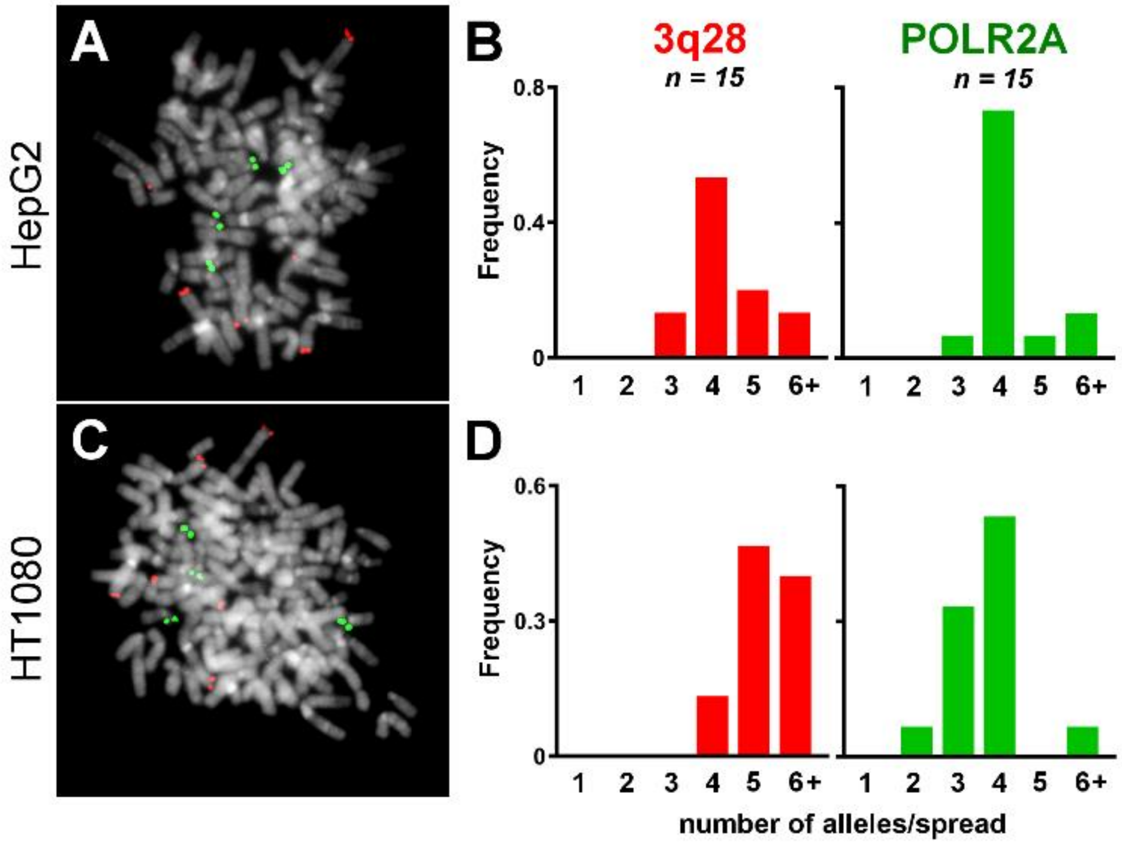

Figure S3. Determination of gene copy number. (A, C) Representative FISH results on metaphase spreads from cell lines that were used in this study. Red: signals from a probe against a region near the TFRC gene (3q28, ~4 Mb away). Green: signals from a probe comprising the POLR2A gene. DAPI counterstain. (A) HepG2. (C) HT-1080. **(B, D)** Frequency distribution of the number of doublet signals per metaphase spread for a region near TFRC (red) and POLR2A (green). 15 metaphase spreads were analyzed for each marker. Cells are aneuploid and the majority have 4 copies of each target gene. (B) HepG2. (D) HT-1080.

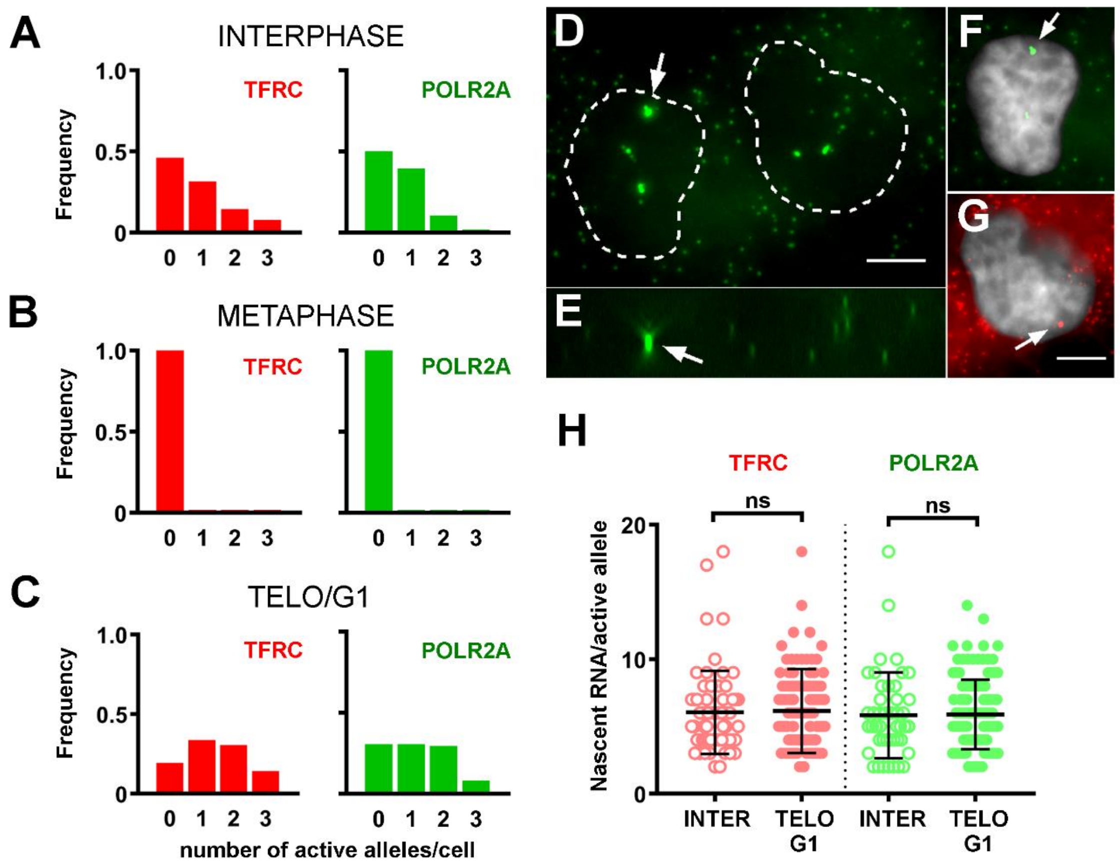

Figure S4. Increased transcription upon mitotic exit in HT-1080 cells. (A-C) Frequency distribution of the number of active alleles per HT-1080 cell for TFRC (red) and POLR2A (green), at interphase (A, total of 76 cells), metaphase (B, total of 42 cells) or telophase/early G1 (C, total of 98 cells). n = 3 experiments. (**D-G)** Representative images of smRNA FISH signals in a pair of daughter cells shortly after mitotic exit (D-E, POLR2A, green) or in individual nuclei (F, POLR2A, green; G, TFRC, red). Shown are *xy* (D, F and G) or *xz* (E) projections of optical sections (thickness of 2 µm for panels D and E, 0.5 µm for F and G). DAPI counterstain in gray. Scale bar, 5 µm. Arrows point to intense nuclear dots which mark putative nascent transcription sites. Notice that the nuclear dots are often found in regions that weakly stain with DAPI. (**H)** Number of nascent RNA molecules per active allele in interphase cells (open circles) or in telophase/early G1 (filled circles). TFRC (red): interphase, total of 64 alleles; telophase/early G1, total of 147 alleles. POLR2A (green): interphase, total

of 46 alleles; telophase/early G1, total of 118 alleles. n = 2 experiments. Mean values (thick lines) ± standard deviation. ns, not significant.

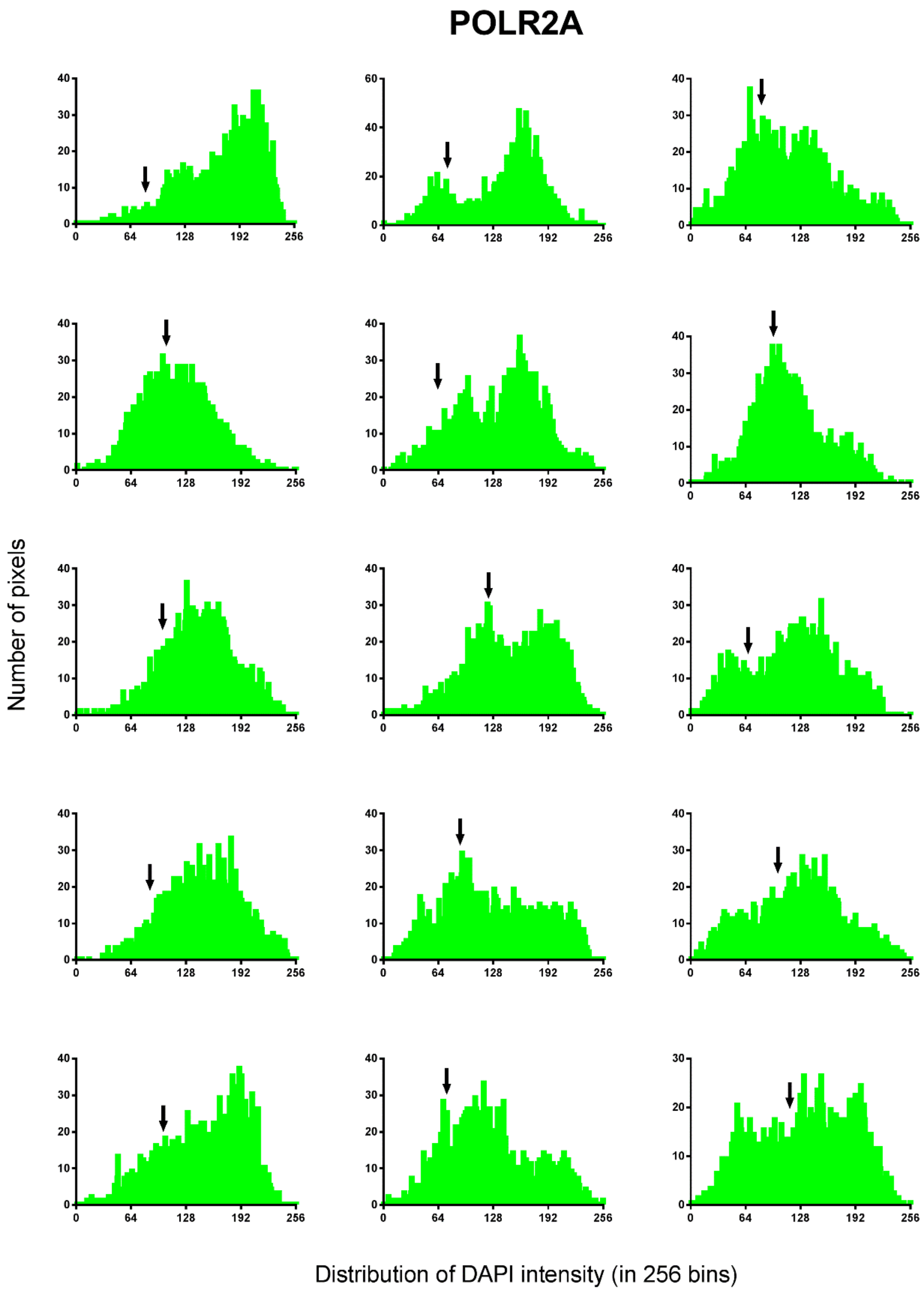

Figure S5. See legend below.

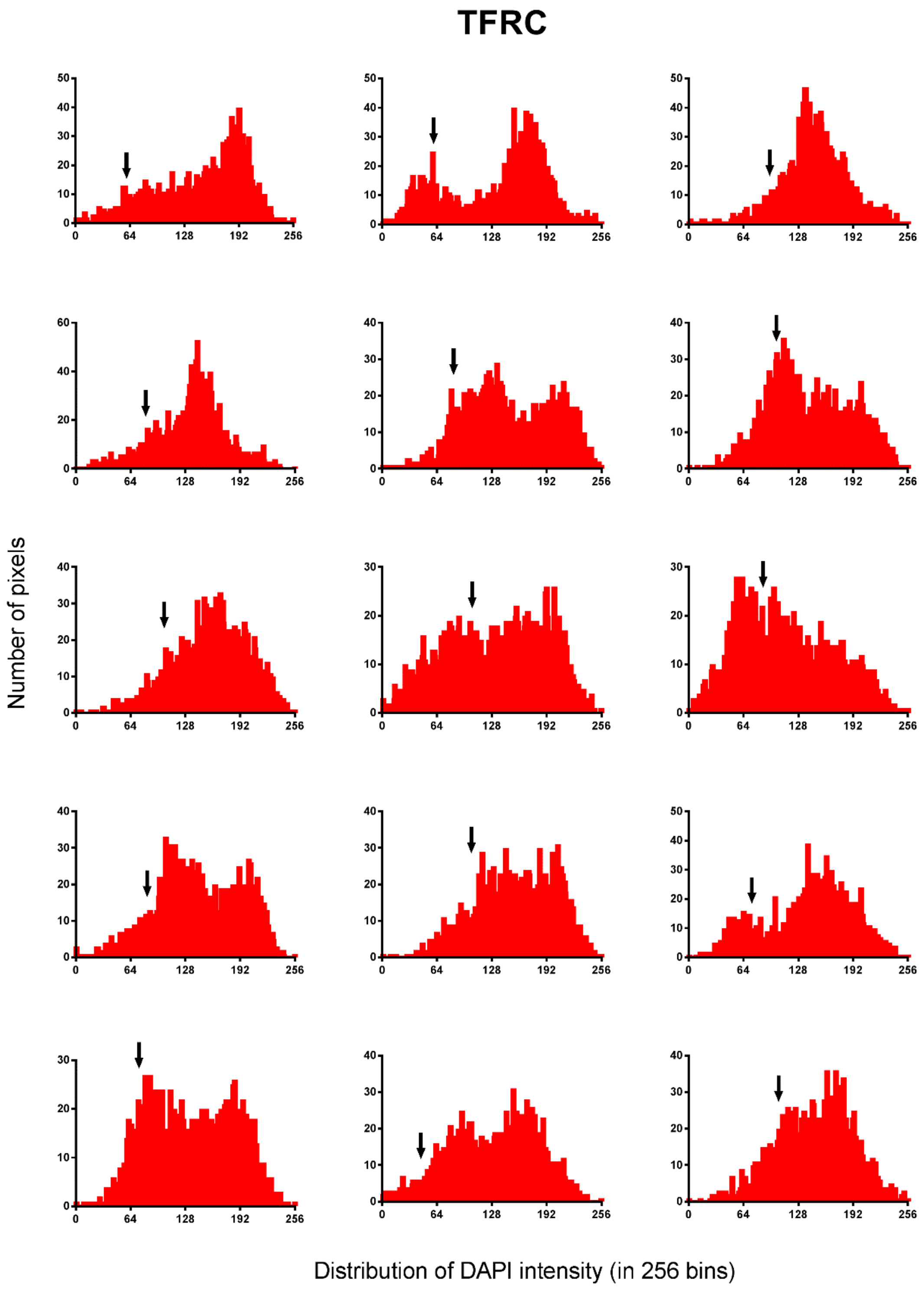

Figure S5. The transcriptional spots that are detected in telophase/early G1 cells are preferentially localized in regions of low DAPI intensity.

Each plot shows the distributions of DAPI intensities in a ~3x3 µm nuclear region containing a given transcriptional spot. The pixel values were distributed in 256 bins of equal size. For

each plot, bin 1 corresponds to the lowest DAPI intensity and bin 256, to the highest. Results are shown for 15 POLR2A spots (green) and 15 TFRC spots (red). Arrows point to the bin that comprises the DAPI intensity at the transcriptional spot. Note that these clearly point to low DAPI intensity in 12/15 cases for POLR2A and in 15/15 cases for TFRC.

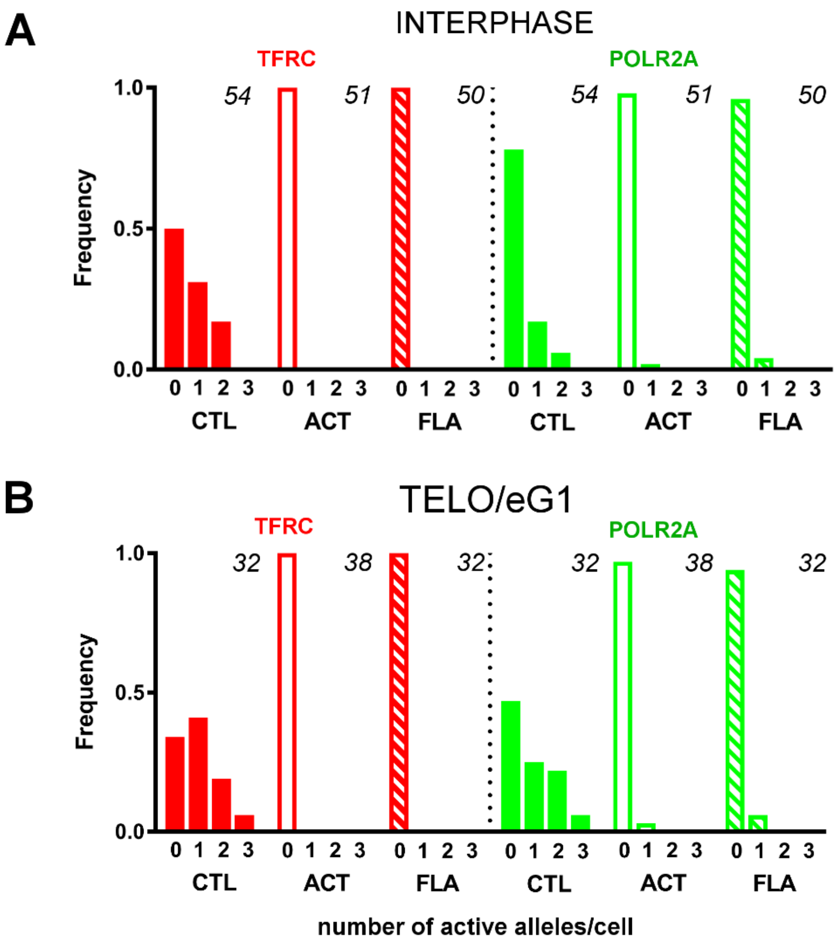

Figure S6. Intense nuclear smRNA FISH signals disappear upon transcriptional inhibition. Cells were treated with an inhibitor of transcriptional elongation (flavopiridol, 1 μM) or with a DNA intercalating compound (actinomycin D, 1.5 μg/ml) for 1 hour before being processed for smRNA FISH. **(A)** Frequency distribution of the number of intense nuclear dots per HepG2 cell in interphase cells for TFRC (red) and POLR2A (green) in control cells (CTL, filled bars, 54 cells), actinomycin D-treated cells (ACT, empty bars, 51 cells) and flavopiridol-treated cells (FLA, dashed bars, 50 cells), n = 1 experiment. **(B)** Frequency distribution of the number of intense nuclear dots per HepG2 cell in telophase/early G1 cells for TFRC (red) and POLR2A (green) in control cells (CTL, filled bars, 32 cells), actinomycin D-treated cells (ACT, empty bars, 38 cells) and flavopiridol-treated cells (FLA, dashed bars, 32 cells), n = 1.

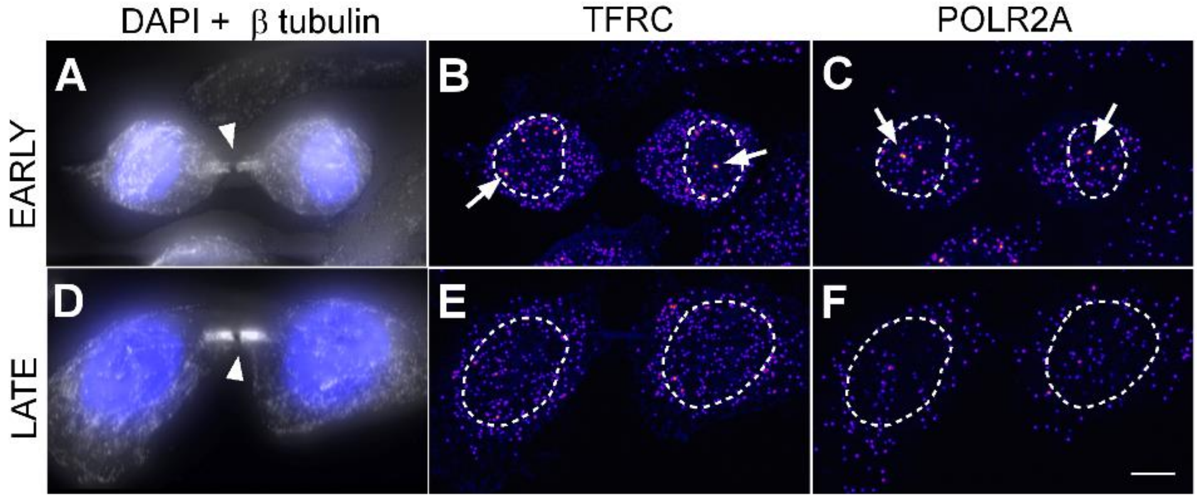

Figure S7. Transcriptional spikes occur early in the cell cycle. Representative images of HepG2 cells co-stained for β-tubulin (left panels), TFRC RNA (middle panels) and POLR2A RNA (right panels). Arrowheads point to abscission midbodies. Arrows point to intense nascent transcriptional spots. The contours of the nuclei are marked by dotted lines. The smRNA FISH signals are shown according to the 'fire' color scheme, i.e. strong signals in yellow/white and weak ones in blue. Shown are pseudo-3D view for β-tubulin and maximum intensity projections for the smRNA FISH signals. **(A)** 'Early' telophase/G1 cells show intense transcriptional spots for both genes. **(B)** Daugther cells that are still joined by a midbody but show no signs of nascent transcription of either genes. Scale bar, 5 μm.

SEQUENCE	Probe name	Probe number	Probe pos	Percent GC
**agttgggaggaaaaagccgg**	hPOLR2A_1	1	270	55,00%
**tcactacaaaaagcctgcgc**	hPOLR2A_2	2	347	50,00%
**gactcaggactccgaactgg**	hPOLR2A_3	3	456	60,00%
**agacattcgcttcagttcat**	hPOLR2A_4	4	485	40,00%
**tcgtctctgggtatttgatg**	hPOLR2A_5	5	519	45,00%
**cagttcaatgtggccaaagt**	hPOLR2A_6	6	650	45,00%
**gggttgttagagtccacaag**	hPOLR2A_7	7	738	50,00%
**aggtcgtagacatgtgtgag**	hPOLR2A_8	8	820	50,00%
**ctgagagtcctcattaacgt**	hPOLR2A_9	9	1010	45,00%
**ttgatcttcacgatgtcagc**	hPOLR2A_10	10	1240	45,00%
**tctgtcaatgttgaaggggg**	hPOLR2A_11	11	1583	50,00%
**agtcaatgcgatcaccattg**	hPOLR2A_12	12	1665	45,00%
**tgtacggagttgtcacacta**	hPOLR2A_13	13	1857	45,00%
**ggaacatcaggaggttcatc**	hPOLR2A_14	14	2079	50,00%
**gagggagaagatttgcttgc**	hPOLR2A_15	15	2162	50,00%
**agaagaggcgagtgatgtca**	hPOLR2A_16	16	2385	50,00%
**gatgaggagccagttgttaa**	hPOLR2A_17	17	2426	45,00%
**tagtgttctgaatgtcctgg**	hPOLR2A_18	18	2483	45,00%
**tcgatgacctctattacgtc**	hPOLR2A_19	19	2533	45,00%
**gcatcgttaagaatgcggtt**	hPOLR2A_20	20	2623	45,00%
**agacagggatttctgagcag**	hPOLR2A_21	21	2663	50,00%
**gacctgggagatgttaatct**	hPOLR2A_22	22	2732	45,00%
**cggtgcttgaagccaaatgg**	hPOLR2A_23	23	2781	55,00%
**aggtaggagttctccacaaa**	hPOLR2A_24	24	2863	45,00%
**cttgttggaaggcttaagcg**	hPOLR2A_25	25	3122	50,00%
**caactcgttctggatgtgtg**	hPOLR2A_26	26	3236	50,00%
**ttcttgctcaattccttgac**	hPOLR2A_27	27	3436	40,00%
**caggtggatgttgaagagca**	hPOLR2A_28	28	3515	50,00%
**ggaaatgttgatgagctcct**	hPOLR2A_29	29	3773	45,00%
**taagcgaaggagtctttggc**	hPOLR2A_30	30	3798	50,00%
**atcttttcagcaatctgctc**	hPOLR2A_31	31	4084	40,00%
**catatctgtcagcatgttgg**	hPOLR2A_32	32	4268	45,00%
**ccgtgatgatgatcttcttc**	hPOLR2A_33	33	4350	45,00%
**tctccacaatgtcattggac**	hPOLR2A_34	34	4473	45,00%
**ggagccatcaaaggagatga**	hPOLR2A_35	35	4553	50,00%
**cacacaagagagccaagtgt**	hPOLR2A_36	36	4587	50,00%
**ctggcccagcatgatattct**	hPOLR2A_37	37	4753	50,00%
**aataagagggactctggggt**	hPOLR2A_38	38	5226	50,00%
**ctatagttgggactggttgg**	hPOLR2A_39	39	5292	50,00%
**gaatagctgggtgatgttgg**	hPOLR2A_40	40	5773	50,00%
**ggaaggggagtaacttggtg**	hPOLR2A_41	41	5801	55,00%
**tataggttggagactgtggt**	hPOLR2A_42	42	5835	45,00%

**gctgggactgtaagaaggac**	hPOLR2A_43	43	5929	55,00%
**ggtgggtgaatatttgggac**	hPOLR2A_44	44	5992	50,00%
**ggagatgttggggagtattt**	hPOLR2A_45	45	6060	45,00%
**ctagtaggtgagtacttggg**	hPOLR2A_46	46	6120	50,00%
**aacgggatccagaagttcac**	hPOLR2A_47	47	6450	50,00%
**acccctaagttaaaataccc**	hPOLR2A_48	48	6616	40,00%

Table S1. Oligonucleotide sequences of the smRNA FISH probe against human POLR2A. Shown in this table are the sequences of individual labeled oligonucleotides (20-mer, from 5' to 3'), the oligonucleotide identification and number, its position on the mature mRNA and its GC contents. The Ensembl Gene ID for human POLR2A is ENSG00000181222.

SEQUENCE (5'->3')	Probe name	Probe no.	Probe pos	Percent GC
			in intron 1	
cataagcagcgagaaagcgc	hPOLR2A_i1_1	1	74	55.0%
ctgctcaactctttgcaaaa	hPOLR2A_i1_2	2	168	40.0%
catctcggacaaagcgctac	hPOLR2A_i1_3	3	190	55.0%
ctggagtgtgaaatcagtca	hPOLR2A_i1_4	4	248	45.0%
atcgcctttaacgtcggtaa	hPOLR2A_i1_5	5	270	45.0%
gtaaagactccctaggattc	hPOLR2A_i1_6	6	295	45.0%
ggtttggcttcttaaccaaa	hPOLR2A_i1_7	7	318	40.0%
cagaagaggaggaagctacc	hPOLR2A_i1_8	8	340	55.0%
cttagcgccacaagggaaaa	hPOLR2A_i1_9	9	1456	50.0%
cttaactccattctttccag	hPOLR2A_i1_10	10	1492	40.0%
ggtctctatccacaaacagc	hPOLR2A_i1_11	11	1514	50.0%
tgaactttctcccagcaaaa	hPOLR2A_i1_12	12	1536	40.0%
acgttctgactcctgacata	hPOLR2A_i1_13	13	1558	45.0%
ctgttctagctgttcttaca	hPOLR2A_i1_14	14	2830	40.0%
ttcactctgcatacttctga	hPOLR2A_i1_15	15	5327	40.0%
cgtaaatacgttcccacttt	hPOLR2A_i1_16	16	5358	40.0%
tttgtcaacagtgtcccata	hPOLR2A_i1_17	17	5796	40.0%
ccagagatgactctggtaga	hPOLR2A_i1_18	18	5818	50.0%
atcgaagcccaacaatccta	hPOLR2A_i1_19	19	5842	45.0%
aactgcaagacatgcagagc	hPOLR2A_i1_20	20	5865	50.0%
ggtccctcaaaatacagaca	hPOLR2A_i1_21	21	5892	45.0%
ctacccaaaattgaaccgtc	hPOLR2A_i1_22	22	5914	45.0%
caacaagaggcaccttactc	hPOLR2A_i1_23	23	5952	50.0%
atacactctgacccctaaga	hPOLR2A_i1_24	24	6012	45.0%
aattcaaagacccctccgta	hPOLR2A_i1_25	25	6037	45.0%
cttctaacagcaaggacaac	hPOLR2A_i1_26	26	6074	45.0%
caaagctctagtaccaaccc	hPOLR2A_i1_27	27	6135	50.0%
aaattttgtgcacacgctgg	hPOLR2A_i1_28	28	6157	45.0%
gaaatccagcaccctctttc	hPOLR2A_i1_29	29	6181	50.0%
aataggggtcaactactgcg	hPOLR2A_i1_30	30	6204	50.0%
gcattccatgatagacagtt	hPOLR2A_i1_31	31	6234	40.0%
caatgtagcccacactctac	hPOLR2A_i1_32	32	6760	50.0%
caaggatcatcttctcactc	hPOLR2A_i1_33	33	6782	45.0%
tccctcacaagattataggt	hPOLR2A_i1_34	34	6812	40.0%
ctcccacatttatgttctaa	hPOLR2A_i1_35	35	7138	35.0%
acagctttatttggtaccac	hPOLR2A_i1_36	36	7172	40.0%
gcaaagctaacaaggtcctt	hPOLR2A_i1_37	37	7219	45.0%
ggagctgtcattgaatctca	hPOLR2A_i1_38	38	7266	45.0%
cctgaggccacaataacaaa	hPOLR2A_i1_39	39	7289	45.0%
tgggttctctttgggttctg	hPOLR2A_i1_40	40	7311	50.0%
ttaaacacttgagggggtgg	hPOLR2A_i1_41	41	7341	50.0%
ctcactgcctacaggataaa	hPOLR2A_i1_42	42	7363	45.0%

**cattttaaattagcccccaa**	hPOLR2A_i1_43	43	7403	35.0%
**tccatcaccaagttcacgaa**	hPOLR2A_i1_44	44	7744	45.0%
**tgtcctagacagcagacacg**	hPOLR2A_i1_45	45	7794	55.0%
**tgctttcaggctttctgaga**	hPOLR2A_i1_46	46	7827	45.0%
**ccacccttctaatgactaat**	hPOLR2A_i1_47	47	7849	40.0%

Table S2. Oligonucleotide sequences of the smRNA FISH probe against the first intron of human POLR2A. Shown in this table are the sequences of individual labeled oligonucleotides (20-mer, from 5' to 3'), the oligonucleotide identification and number, its position relative to the first nucleotide of the intron and its GC contents. The Ensembl Gene ID for human POLR2A is ENSG00000181222.
